# Identification and genetic characterization of hepacivirus and pegivirus in commercial equine serum products in China

**DOI:** 10.1371/journal.pone.0189208

**Published:** 2017-12-07

**Authors:** Gang Lu, Ji Huang, Qiliang Yang, Haibin Xu, Peixin Wu, Cheng Fu, Shoujun Li

**Affiliations:** 1 College of Veterinary Medicine, South China Agricultural University, Guangzhou, Guangdong Province, People’s Republic of China; 2 Guangdong Provincial Key Laboratory of Prevention and Control for Severe Clinical Animal Diseases, Guangzhou, Guangdong Province, People’s Republic of China; 3 Guangdong Technological Engineering Research Center for Pet Guangzhou, Guangdong Province, People’s Republic of China; Kliniken der Stadt Köln gGmbH, GERMANY

## Abstract

Equine hepacivirus (EqHV), equine pegivirus (EPgV) and Theiler’s disease-associated virus (TDAV) are three novel equine viruses in the family *Flaviviridae*. EqHV and EPgV have been identified to circulate in the equine population worldwide, whereas TDAV has not been detected in equines since the first reported case. To date, no studies have focused on investigating EqHV, EPgV and TDAV in commercial equine sera or equine blood products in China. Considering the potential threat of EqHV, EPgV and TDAV to biosecurity and considering their possible influences on research results, equine sera for cell culture propagation and pregnant mare serum gonadotropin (PMSG) were purchased from different companies in China and investigated for EqHV, EPgV and TDAV in this study. By performing nested PCR or two rounds of PCR targeting the viral NS3 gene, four serum samples were confirmed to be EqHV-, EPgV-, or TDAV-RNA positive; all of the PMSG samples were negative for these three viruses. Subsequent sequencing results indicated that the serum samples contained multiple viral variants of EqHV, EPgV or TDAV, and a genetic analysis based on the partial NS3 gene of the three equine viruses was performed. Our study is the first to confirm the presence of EqHV, EPgV and TDAV in equine sera for cell culture propagation that is commercially available in China and provides the first demonstration of the presence of TDAV in China.

## Introduction

According to International Committee on Taxonomy of Viruses (ICTV), the family *Flaviviridae* includes the following four genera: *Flavivirus*, *Hepacivirus*, *Pestivirus* and the newly proposed genus *Pegivirus*. Recently, three novel viruses belonging to the family *Flaviviridae* have been detected in the equine population, namely, equine hepacivirus (EqHV)[1], equine pegivirus (EPgV)[2] and Theiler’s disease-associated virus (TDAV)[3]. As a newly identified member of the *Hepacivirus* genus, EqHV was first reported in the USA in 2012 and is the first identified non-primate hepacivirus (NPHV) after canine hepacivirus virus (CHV)[1, 4]. Phylogenetic analyses of hepacivirus sequences from various species have indicated that EqHV clusters together with CHV and that this virus is genetically closest to Hepatitis C virus (HCV). Both EPgV and TDAV are classified into the genus *Pegivirus* together with pegiviruses from humans[5], monkeys[6], bats[7] and rodents[8]. Until now, whether EqHV infection is associated with liver disease remains unclear. However, it has been reported that horses infected with EqHV could developed severe hepatitis and chronic wasting[9, 10]. EPgV and TDAV were first discovered in horses in the USA in 2013[2, 3]. In the first report of EPgV, two serum samples collected from horses with elevated liver enzyme levels were identified to be EPgV-RNA positive[2]. Nucleotide and translated amino-acid sequence analyses indicated that EPgV is genetically similar to human pegiviruses (HPgV), as well as to simian and bat pegiviruses[7, 11]. Further studies indicate that EPgV is associated with persistent viremia in equines, and also may be a cause or contributory factor in equine hepatopathy[2, 12]. TDAV is so named because this virus might be a causative agent of Theiler’s disease, which is an acute hepatitis in horses associated with the administration of equine blood products[3]. TDAV is closer genetically to the GB virus than are HCV and NPHV. By detecting viral RNA and/or antibody against viral proteins in serum or plasma, EqHV and EPgV have been identified in the equine population in American, European and Asian countries[10, 12–18]. However, equines infected with TDAV have not been identified in other studies worldwide since the first reports[12, 13, 16, 17].

Human hepacivirus and pegivirus can be transmitted via blood or blood products[19, 20]. Among the three novel equine viruses in the family *Flaviviridae*, EqHV and TDAV have been reported as transmittable by inoculation with plasma or the administration of equine blood products[3, 21]. The transmission route of EPgV has not been studied. However, considering that human pegivirus can be transmitted via blood and from blood donors to recipients[22, 23], the possibility of the transmission of EPgV via equine blood or blood products cannot be ruled out. Equines have been used to produce equine sera for cell culture and other blood products. Therefore, the biosecurity of commercially available equine sera or blood products warrants attention. Moreover, commercial available equine sera have been produced for cell culture propagation, and exogenous EqHV, EPgV and TDAV contained in such sera might influence research studies. It is therefore important to detect these viruses before using equine sera for cell culture propagation or equine blood products.

In 2016, Postel A *et al* reported that high proportions of equine sera for cell culture propagation commercially available in Germany were contaminated with EqHV (5/6), EPgV (6/6) and TDAV (3/6)[17]. Previously, we reported that EqHV and EPgV circulate in the equine population in China, with a prevalence of 6/177 and 2/177, respectively[13]. However, whether commercial equine sera for cell culture propagation or equine blood products in China are contaminated with EqHV, EPgV or TDAV is not known. Considering the importance of determining whether such contamination exists, we purchased equine sera for cell culture propagation and equine blood products that are commercially available in China, tested for EqHV, EPgV and TDAV in the samples, and performed a genetic analysis of the detected three viruses in this study.

## Materials and methods

### Serum product information

To test for possible contamination with EqHV, EPgV and TDAV, equine sera for cell culture propagation that are commercially available in China (n = 9) were included in our study (Table 1); among the samples, eight samples were horse serum and one sample was donkey serum. The geographic origins of the samples were China (seven samples from domestic equines) and the USA (two samples from horses). Pregnant mare serum gonadotropin (PMSG) is a gonadotropic hormone obtained from the serum of pregnant mares and is used to induce follicular growth, estrus and ovulation in various animals by direct injection[24, 25]. A total of six PMSG products were obtained from different companies in China and used for the detection of EqHV, EPgV and TDAV. The serum and PMSG samples were stored at -70°C immediately after purchase.

**Table 1:** Information on the equine sera and the identification of viral RNA by PCR in this study.

Serum information			Viral RNA identified by PCR		
Serum ID	Specification	Geographic origin	EqHV	EPgV	TDAV
A	Horse Serum	China	+	-	+
B	Donkey Serum	China	-	-	-
C	Horse Serum, sterile	China	-	-	-
D	Horse Serum	China	-	-	-
E	Horse Serum	China	-	-	-
F	Horse Serum	China	+	+	+
G	Horse Serum	China	-	-	-
H	Horse Serum,sterile	USA	+	-	+
I	Horse Serum,sterile	USA	+	+	+

+, PCR positive; -, PCR negative.

### RT-PCR and sequencing

Total RNA from 200-uL samples was prepared with the MiniBEST Viral RNA/DNA Extraction Kit (Takara, Dalian, China) according to the manufacturer's protocol. Two microliters of RNA were used for random-primed reverse transcription using HiScript®II 1st Strand cDNA Synthesis Kit (Vazyme, Nanjing, China), and a target sequence in the nonstructural 3 protein (NS3) region of the EqHV genome was amplified by nested PCR using two sets of degenerate primers as described previously[13]. To determine whether the samples contained EPgV and TDAV genome, one primer set targeting a conserved section in the NS3 coding region of both viruses was used in two rounds of PCR. Finally, the second-round product detected in a 1.5% agarose gel with a band of 173 bp and 355 bp was considered EqHV and EPgV and/or TDAV positive, respectively.

The nucleic acid contained in the agarose gel was then extracted and cloned into the PLB vector using the Lethal Based Fast Cloning Kit (TianGen, Beijing, China). The construct was transformed into *E*. *coli*, and the transformed bacteria were identified using PCR. A total of twenty positive *E*. *coli* clones for each virus were subsequently sent for Sanger sequencing using primers (PLB-F: CGACTCACTATAGGGAGAGCGGC, PLB-R: AAGAACATCGATTTTCCATGGCAG) (BGI, Guangzhou, China).

### Sequence analysis and phylogenetic tree construction

Using the BLASTN tool in the NCBI database, the sequence data were analyzed to confirm whether the sequenced *E*. *coli* clones contained the nucleotide sequence of the partial viral NS3 region of EqHV, EPgV and or TDAV. Then, the raw sequence data of the partial viral NS3 region of the EqHV, EPgV and TDAV strains in this study were edited and aligned with those of other viruses acquired from the GenBank database by the Bioedit 7.0.9.0 software program. Under the HKY substitution model, a Maximum Clade Credibility (MCC) phylogenetic tree based on the differences among the viral sequences was constructed using Beast v1.8.2, and an evolutionary analysis of the identified EqHV, EPgV and TDAV sequences was performed.

The viral sequences obtained in this study are available online in the GenBank database under accession nos. KY922911-KY922953.

## Results

### Identification of EqHV, EPgV and TDAV in commercial equine serum products in China

To investigate EqHV, EPgV and TDAV in commercial equine serum products from China, a total of nine cell-culture equine sera and six PMSG products were purchased companies in China and USA (Table 1). The presence of EqHV, EPgV and TDAV in the equine serum products was determined by performing nested PCR or two rounds of PCR with primers targeting the viral NS3 gene. The PCR and subsequent sequencing and BLAST results indicated that among the serum samples originating from donor equines from China, one sample (serum A) was both EqHV- and TDAV-RNA positive, and a second sample (serum F) contained the genomes of all three viruses. Of the two sera with a geographic origin of the USA, one was EqHV- and TDAV-RNA positive (serum H), and the other was EqHV-, EPgV-, and TDAV-RNA positive (serum I). No EqHV, EPgV, or TDAV RNA was identified in serum samples B-E or G, which were all produced from native equines in China. In total, the numbers of cell culture sera commercially available in China that were positive for EqHV, EPgV, and TDAV were 4/9, 2/9, and 4/9, respectively. This is the first report of the identification of EqHV, EPgV and TDAV in commercial cell cultures of equine sera in China. In contrast, no EqHV, EPgV, or TDAV RNA was detected in the commercial PMSG products from China.

### Genetic and phylogenetic characterization of EqHV, EPgV and TDAV in commercial equine serum products from China

The sequencing results of twenty positive clones for each virus of EqHV, EPgV and TDAV were analyzed. Using the BLAST tool of the NCBI website, nine, four and eleven different EqHV strain sequences were found in sera A, H and F, respectively, namely, A1-A9, F1-F4 and H1-H11, with a similarity of 97.1-99.84%, 98.84-99.42% and 85.54-99.42%, respectively, at the nucleotide level and a similarity of 96.49-100%, 96.49-100% and 96.49-100%, respectively, at the amino-acid level. Two and one EPgV strain sequences were found in sera F and I, respectively, namely, F5 and I1-I2 (with a similarity of 99.15% and 100% at the nucleotide and amino acid level, respectively). Three, six, three and three TDAV strain sequences were found in sera A, F, H and I, respectively, with a similarity of 98.87-99.15%, 99.43%, 99.15-99.71% and 99.43%, respectively, at the nucleotide level and of 96.61-98.3%, 99.15-100%, 99.15-100% and 100%, respectively, at the amino-acid level.

**Fig 1:**
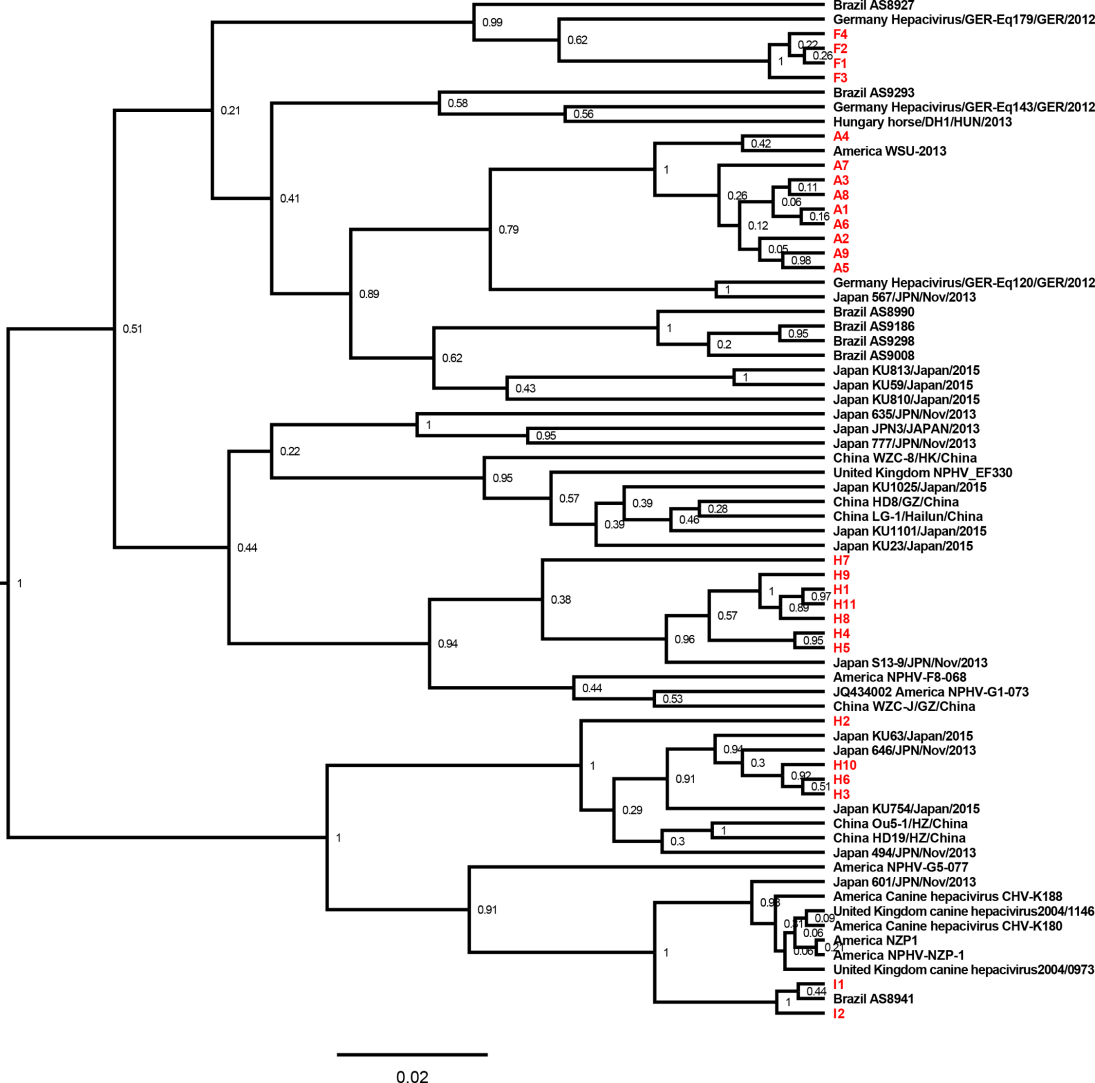
Phylogenetic analysis of EqHV strains in commercial equine serum products in China. A phylogenetic tree based on the partial NS3 gene of virus strains was constructed using Beast v1.8.2. Posterior probability values are indicated at the nodes. The strain name and the country where the strain was identified are shown. The strains confirmed in this study are labeled in red.

As indicated by the phylogenetic tree established based on the partial NS3 gene sequences of the EqHV strains (Fig 1), the EqHV strains identified in equine sera used for cell culture propagation that are commercially available in China had a different evolutional relationship with other reported EqHV strains. The phylogenetic analysis indicated that the EqHV strains identified in serum A had a close relationship between each other, and were clustered together with an American EqHV strain, WSU-2013. The four EqHV strains in serum F were clustered together with two EqHV strains identified in Germany and Brazil (Hepacivirus/GER-Eq179/GER/2012, AS8927). It was noted that the eleven EqHV strains in serum H had a divergent evolutional pattern based on the partial NS3 gene sequences. The strains H1, 4, 5, 7, 8, 9 and 11 were clustered together, whereas H2, 3, 6 and 10 were clustered in another group. The EqHV strains in serum I (I1, 2) was close to one Brazilian EqHV strain, AS8941.

**Fig 2:**
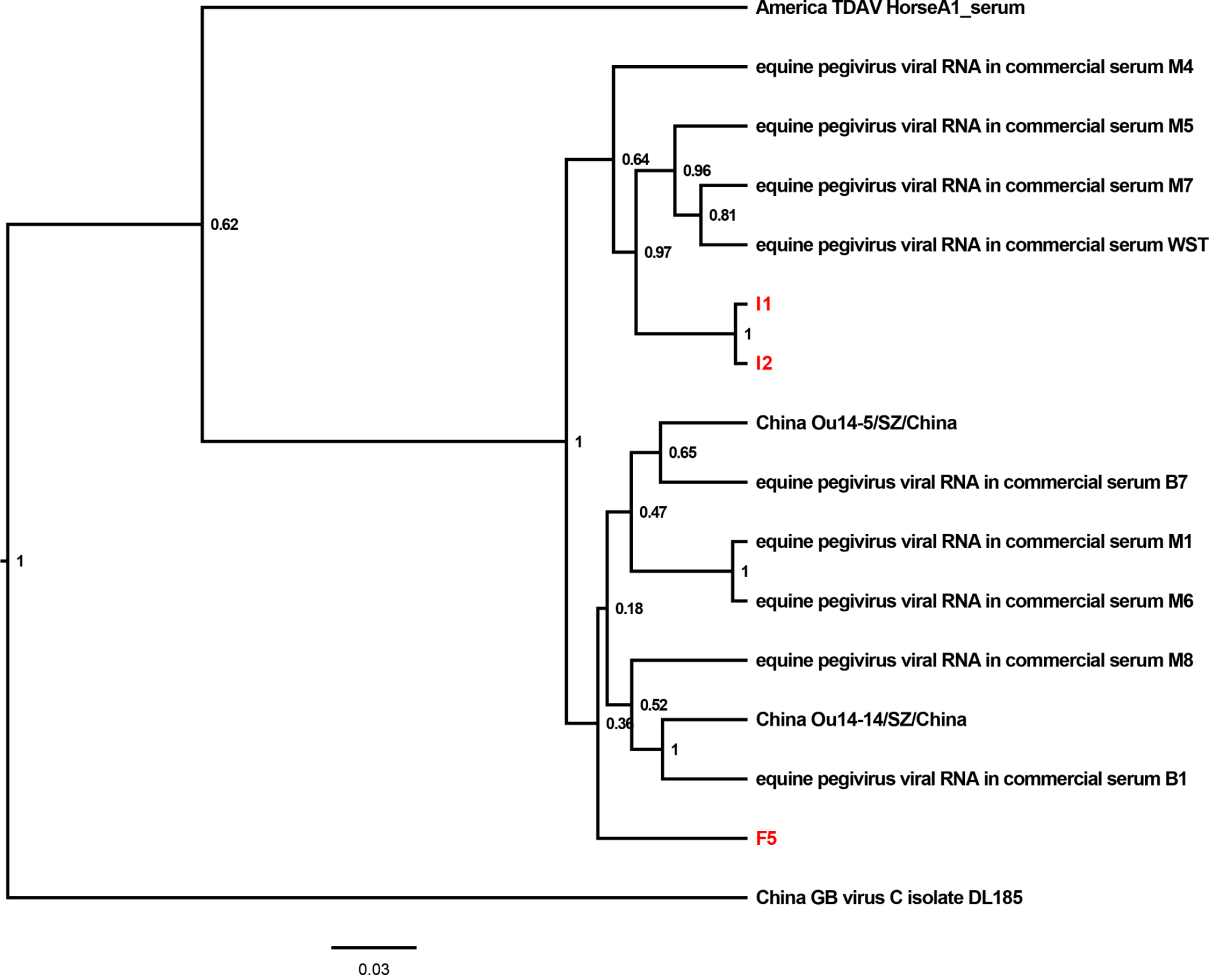
Phylogenetic analysis of EPgV strains in commercial equine serum products in China. A phylogenetic tree based on the partial NS3 gene of virus strains was constructed using Beast v1.8.2. Posterior probability values are indicated at the nodes. The strain name and the country where the strain was identified are shown. The strains confirmed in this study are labeled in red.

The BLAST results for the partial NS3 sequences of the three EPgV strains identified in this study (I1, I2, F5) yielded hits for EPgV RNA in serum as well as two other field strains of EPgV (Ou14-5/SZ/China, Ou14-14/SZ/China) from equines in China. The phylogenetic analysis revealed that I1, I2 and F5 were grouped together with EPgV strains from commercial equine sera reported in other studies and field strains of EPgV reported previously in equines, whereas GB virus C and TDAV were clustered together as an outgroup (Fig 2). In addition, I1 and I2 were clustered with each other, and F5 was clustered together with the two field EPgV strains of Ou14-5/SZ/China and Ou14-14/SZ/China.

**Fig 3:**
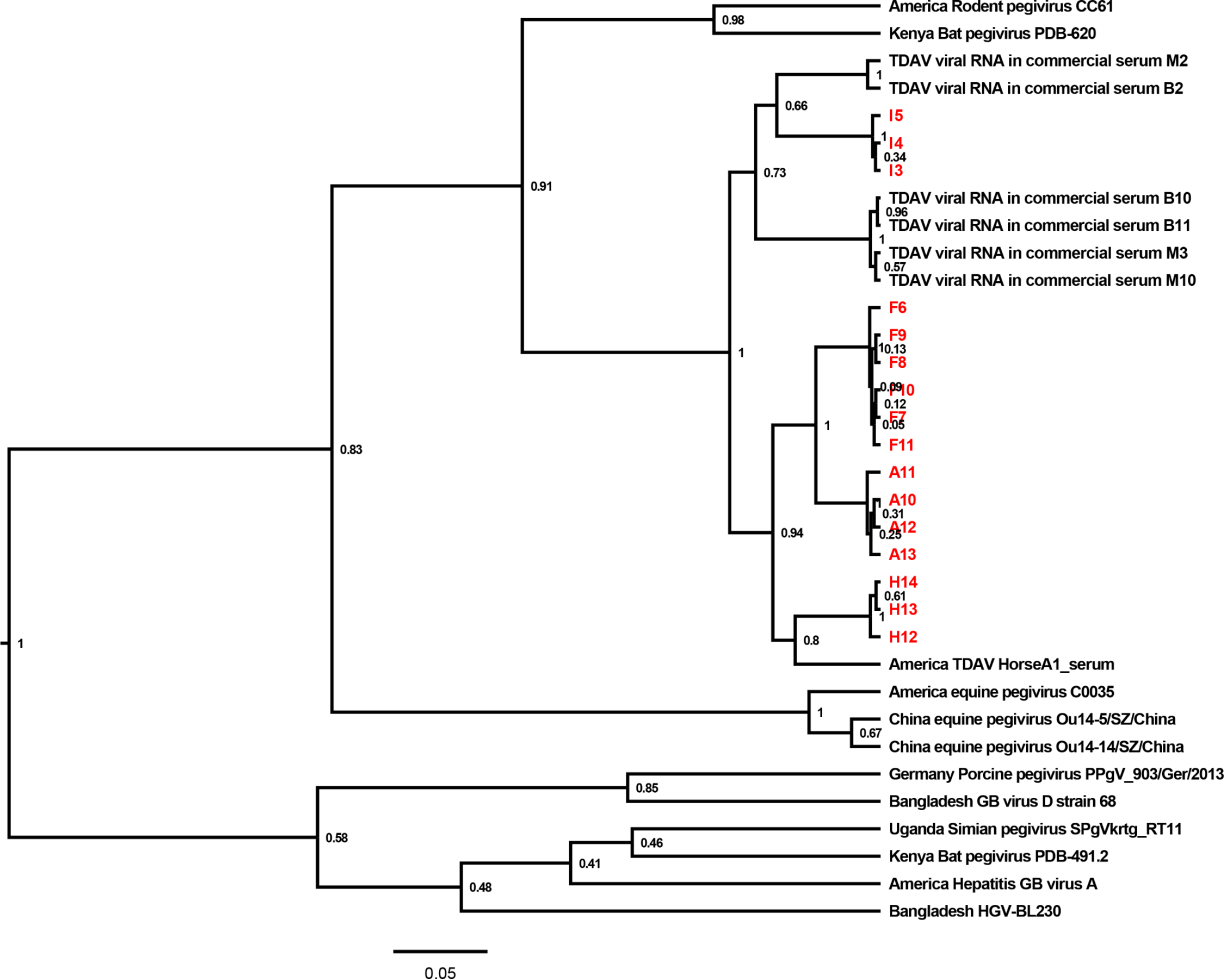
Phylogenetic analysis of TDAV strains in commercial equine serum products in China. A phylogenetic tree based on the partial NS3 gene of virus strains was constructed using Beast v1.8.2. Posterior probability values are indicated at the nodes. The strain name and the country where the strain was identified are shown. The strains confirmed in this study are labeled in red.

Using the partial NS3 sequences of TDAV strains detected in commercial equine serum used for cell culture propagation and present in the equine population, the pegivirus from other animals and GB viruses, a phylogenetic analysis was performed (Fig 3). TDAV strains detected in our study clustered with the TDAV strains previously detected in the equine population, whereas the pegivirus from other animals and the GB viruses formed the outgroup. The TDAV strains I3, I4 and I5 were clutsered together with other TDAV strains reported in commercial sera by Postel A et al, whereas the remaining 13 TDAV strains detected in this study (F6-F11, A10-A13, H12-A14) were grouped with each other, clustered together with the first reported TDAV strain of HorseA1_serum identified in the USA in 2013.

## Discussion

Between 2014 and 2015, we performed a surveillance study to investigate the prevalence of three novel equine viruses, EqHV, EPgV and TDAV, in the equine population of China[13]. A total of 177 serum samples were collected, among which six and two samples were found to be EqHV- and EPgV-RNA positive, respectively. Samples containing the RNA of both viruses were not detected, and all of the samples were TDAV-RNA negative. In 2016, Postel A *et al* detected EqHV, EPgV and TDAV RNA in six samples of commercial equine sera in Germany using the triplex real time RT-PCR method, and they found that all of the studied commercially available equine sera purchased for cell culture propagation were EqHV-, EPgV-, or TDAV-RNA positive[17]. To date, no studies have investigated EqHV, EPgV and TDAV in commercial equine sera or equine blood products in China. Considering the potential threat of EqHV, EPgV and TDAV to biosecurity and their possible influences on research results, we purchased nine equine sera used for cell culture propagation and six equine PMSG products that are commercially available in China and tested them for the presence of EqHV, EPgV and TDAV. All of the PMSG products were identified as EqHV-, EPgV- and TDAV-RNA negative, whereas 4/9, 2/9, and 4/9 of the equine sera samples were found to be EqHV-, EPgV- and TDAV-RNA positive, respectively (Table 1). The numbers of samples of commercial equine sera that were positive for EqHV, EPgV and TDAV were lower than values of 5/6, 6/6 and 3/6, respectively, reported by Postel A *et al*[17]. However, the different detection limits and sensitivity of the test methods used by Postel A *et al* (real time RT-PCR) and by us (nested PCR and two rounds of PCR) might have contributed to these differences. In addition, due to the limited amount of information on the genome of these three viruses, the target range of the primers used in the present study might not be very highly conserved. Accordingly, the true prevalence of EqHV, EPgV and TDAV in commercial equine sera used for cell culture propagation in China requires further research. Nearly all viruses are detected in two samples from USA. Because a limited number of samples detected in this study, this result does not mean much more prevalent of EqHV, EPgV and TDAV in USA than that in China. This needs further study. It was noted that no viral RNA of EqHV, EPgV and TDAV was detected in the PMSG products. It might be caused by RNA degradation during PMSG production and processing. However, due to the limited sample number in this study, whether the PMSG products could be contaminated with EqHV, EPgV and TDAV is not clear. More large-scale investigations may help solve this problem. Our study provides the first report of the identification of EqHV, EPgV and TDAV in commercial equine sera used for cell culture propagation in China.

In the present study, different EqHV, EPgV and TDAV genome types were identified in the commercial equine serum samples (Figs 1–3). Viral RNA polymerase has low fidelity, and the pegiviruses and hepaciviruses are considered to propagate with high genetic variability [26–28]. The highly genetically variable characteristics of EqHV, EPgV and TDAV make it challenging to design conserved primers for use in PCR and highlight the importance of continuously monitoring these viruses.

Since 2013, when TDAV was confirmed as a novel equine virus in the USA, surveillance studies conducted among the equine population worldwide have not found further evidence of TDAV in equines. These studies include a previous study by our group on the equine population in China between 2014 and 2015[13]. In contrast, the study by Postel A *et al* and the present study detected TDAV RNA in commercial equine sera used for cell culture propagation in Germany and China, respectively, with high detection rates of 3/6 and 4/9, respectively. The primers and PCR procedure applied in the present study are consistent with those used in previous study[13]. Why TDAV has not been detected worldwide since its first report is not clear. Presently, knowledge of TADV is very limited; even the natural reservoirs and hosts of this virus have not been identified. Moreover, in the GenBank database, there is only one complete genome sequence of TDAV. It is difficult to amplify the complete genome of TDAV using the Sanger sequencing method; use of next-generation sequencing might help solve this problem, as described in the first report of this virus[3]. Our study provides the first evidence of the presence of TDAV in China, but whether TDAV is circulating in equines in China remains unclear. The donor horses of serum A and serum F belonged to two different companies and were located in two distinct reigns in China; however, we cannot not rule out the possibility that the serum was contaminated with exogenous TDAV in the production of the commercial equine serum. Therefore, further research is needed to clarify the prevalence of TDAV in equines in China.

In conclusion, this study provides the first evidence of the presence of EqHV, EPgV and TDAV in equine sera for cell culture propagation that is commercially available in China, and a genetic analysis based on the partial NS3 gene iof the three equine viruses was performed. This study also provides the first demonstration of the presence of TDAV in China; the prevalence of TDAV in equines in China remains unclear. This study emphasizes the importance of monitoring the biosecurity of commercially available equine sera and blood products and of considering the possible influences of EqHV, EPgV and TDAV in commercial available equine sera on research studies.

## Supporting information

S1 Fig RT-PCR result of EqHV, EPgV and TDAV in commercial equine sera for cell culture propagation in China. (TIF)
