# Supplementary figures and images for: Identification and genetic characterization of hepacivirus and pegivirus in commercial equine serum products in China
Source: PLoS One. 2017 Dec 7;12(12):e0189208. doi: 10.1371/journal.pone.0189208 (PMC5720783; doi:10.1371/journal.pone.0189208)

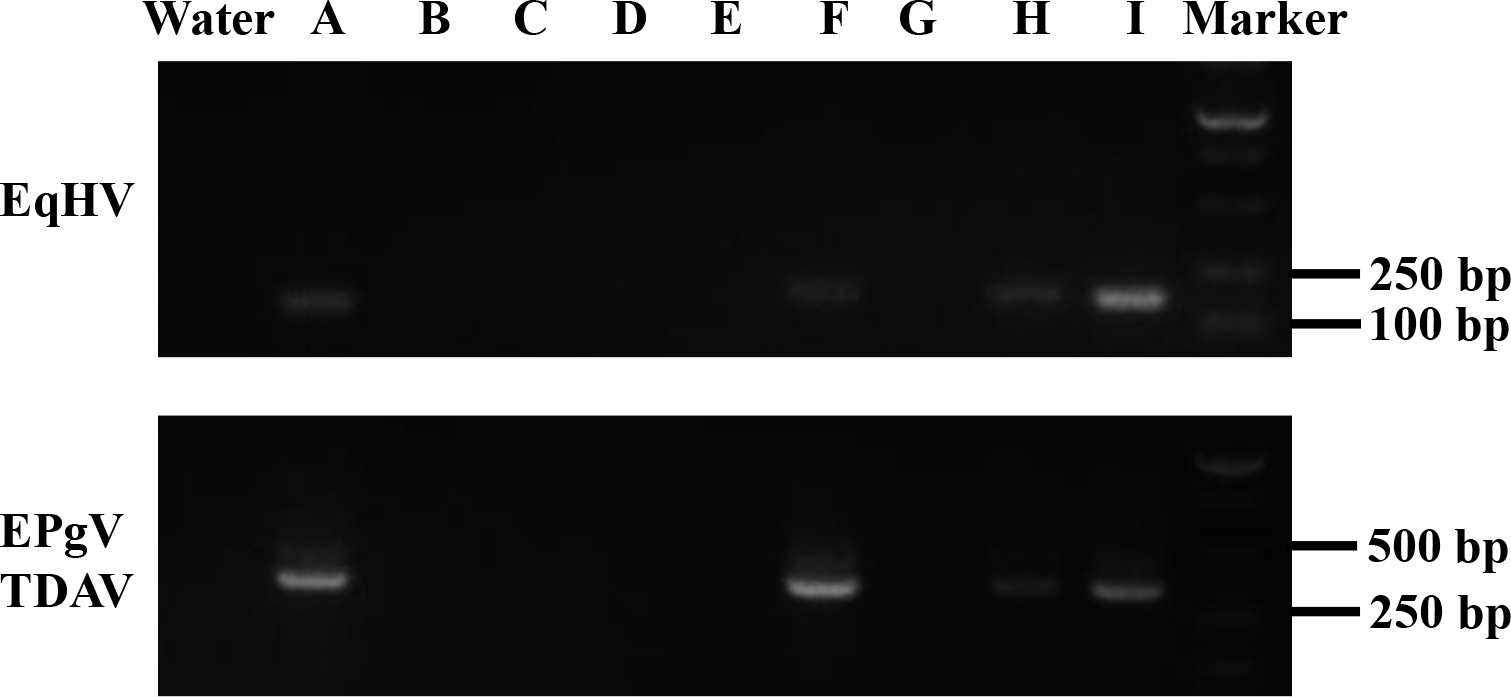

Supplement: S1 Fig — (TIF) [file pone.0189208.s001.tif]
